# Adapting systematic scoping study methods to identify cancer-specific physical activity opportunities in Ontario, Canada

**DOI:** 10.1186/s13643-022-01886-8

**Published:** 2022-01-18

**Authors:** Angela J. Fong, Catherine M. Sabiston, Kaitlyn D. Kauffeldt, Jennifer R. Tomasone

**Affiliations:** 1 Section of Behavioral Sciences, Rutgers Cancer Institute of New Jersey, 195 Little Albany Street, New Brunswick, NJ 08903-2681 USA; 2 Faculty of Kinesiology and Physical Education, University of Toronto, Toronto, Canada; 3 School of Kinesiology and Health Studies, Queen’s University, Kingston, Canada

**Keywords:** Knowledge synthesis, Cancer survivorship, Physical activity, Dissemination and implementation science, Knowledge translation

## Abstract

**Background:**

Identifying cancer-specific physical activity programs and post-secondary courses targeting students in academic settings (i.e., “real world” opportunities) may promote physical activity behaviors among cancer survivors. Using knowledge synthesis methods such as systematic scoping study methods may facilitate knowledge tool development and guide evidence-based practice to improve knowledge transfer. However, identifying these opportunities poses a challenge as systematic scoping study methods have yet to be applied and adapted to this context. Thus, to extend systematic scoping study methods, the purpose of the current investigation is to describe the adaptation of systematic scoping study methods in the context of cancer-specific “real world” opportunities in Ontario, Canada.

**Methods:**

Systematic scoping study methods were adapted to develop a knowledge tool, which was a credible resource website for researchers, clinicians, and survivors. Three search strategies including Advanced Google Search, targeted website search, and consultations with experts were used to identify eligible (e.g., appropriate for cancer survivors, offered in the community) cancer-specific physical activity programs. Only the targeted website search was used to search post-secondary institutions because they are centralized onto one government website.

**Results:**

Fifty-eight programs and 10 post-secondary courses met the eligibility criteria. Relevant data from these opportunities were extracted, charted, synthesized, and uploaded onto the resource website. The most successful search strategy for cancer-specific physical activity programs was the targeted website search followed by Google Advanced Search and consultations with content experts.

**Conclusions:**

Challenges were experienced due to lack of standard reporting among opportunities, bias of potentially relevant records, and changing nature of resulting records. The current investigation demonstrated that systematic scoping study methods can be applied to cancer-specific physical activity programs and post-secondary courses in the context of cancer survivorship in Ontario yielding robust results. The method can be further adapted and updated in future knowledge syntheses in health-related contexts.

**Systematic review registration:**

The systematic scoping review method protocol has not been registered.

**Supplementary Information:**

The online version contains supplementary material available at 10.1186/s13643-022-01886-8.

## Background

As many as 70 to 90% of cancer survivors, defined from the time of diagnosis onward [1], report not meeting physical activity guidelines and thus are not able to accrue associated health benefits [2]. Health benefits may include decreased self-reported depression and anxiety, improved cancer-related fatigue, and health-related quality of life [2–7]. However, additional approaches are needed to promote physical activity among cancer survivors.

The Internet offers promise for promoting physical activity to cancer survivors given its reach, accessibility, and interest among the population for using technology [8–11]. Eighty-one percent of survivors report using the Internet to find health information related to their cancer diagnosis and this trend is likely to increase as infrastructure improves [10, 12]. While there is a large amount of information available online, it can be difficult to assess information quality and identify appropriate cancer-specific physical activity programs [11, 13]. For example, entering the search terms “cancer and exercise program” on Google yields approximately 45,400,000 results. It is unlikely that a cancer survivor would spend the time and effort required to sift through all the results and verify that programs and information are appropriate, safe, and currently available [9]. There is evidence to suggest that the quality of online physical activity information for cancer survivors may vary widely [9, 13]. Lower quality information may curtail efficacy for behavior change among cancer survivors [13] as it may not include comprehensive information, such as strategies for increasing physical activity [14]. One strategy for increasing physical activity is instruction from trustworthy and credible sources including qualified exercise professionals. Similar to the variability of online information, qualified exercise professionals may also have varying educational backgrounds [15]. Again, it may be difficult to discern quality training among these individuals. As a result, identifying available cancer-specific physical activity programs and post-secondary courses may be informative for both cancer survivors and qualified exercise professionals alike. Furthermore, access to information may be associated with increased awareness and use of cancer-specific physical activity programs and post-secondary courses, which may increase cancer survivors’ behavior [16].

Identifying cancer-specific physical activity programs and post-secondary courses targeting students, herein referred to collectively as ‘“real world” opportunities, poses a challenge as knowledge synthesis methodologies, such as systematic scoping study methods, have yet to be applied and adapted to this context. Systematic scoping study methods are operationalized as systematic scoping review methods applied to information beyond peer-reviewed and gray literature. Applying and adapting systematic scoping study methods to these types of “real world” opportunities is necessary for extending the methodology [17]. Extending systematic scoping study methods is important to inform knowledge tool development and for guiding evidence-based practice [17]. A proposed extension is to provide a methodological resource on how to conduct this type of knowledge synthesis because it does not currently exist [18]. Furthermore, reproducibility, defined as operationalized steps of the search, is the cornerstone of many knowledge synthesis methodologies [19]. Elucidating challenges experienced and proposed future directions when conducting the search will increase the reproducibility of the methodology and has pragmatic implications for researchers and clinicians who aim to synthesize health-related “real world” opportunities [18, 20].

While a search strategy including peer-reviewed and gray literature could be used, searching information sources that identify “real world” opportunities are likely to yield current cancer-specific physical activity programs and post-secondary courses [17]. However, searching “real world” opportunities is often less systematic and, therefore, difficult to reproduce [18, 21]. Accordingly, a knowledge synthesis strategy was adapted from Godin and colleagues’ [18] and D’Urzo and colleagues’ [17] recommendations for systematic scoping study methods. Compared to both systematic and scoping reviews, systematic scoping studies search various information sources including websites and community-based initiatives and include consultations with content experts to offer a general overview of existing evidence and identify knowledge gaps that may impact practice [17, 22]. Furthermore, systematic scoping studies extend similar methodologies by including comprehensive recommendations that are reproducible and clear with eligibility criteria to decrease bias [17]. Currently, systematic scoping review methods have been adapted to identify community-based physical activity programs for persons with disabilities [17] and examine school-based breakfast programs in Canada [18]. However, systematic scoping review methods have yet to be applied in the cancer and exercise context to identify “real world” opportunities.

### Context

The impetus for this project was to develop content for a knowledge tool—the Canadian Oncology Rehabilitation and Exercise Network (COREN) website (http://www.icanbeactive.com/ [23]). The mission of COREN is to build a network of professionals to advance knowledge of and access to rehabilitation and exercise information following a cancer diagnosis. The target audiences of the website include researchers, clinicians, and survivors. A search was conducted for cancer-specific physical activity programs and post-secondary courses to be aligned with the COREN mission statement. Cancer-specific physical activity programs, operationalized as community-based exercise, recreation, and sport designed for cancer survivors or a similar clinical population such as older adults, were included for several reasons. First, such programs offer an opportunity for cancer survivors to increase physical activity levels and potentially gain associated health benefits [15, 24]. Programs suitable for older adults were included in the event that no cancer-specific programs could be identified in a particular geographic region. Second, a list of current cancer-specific physical activity programs allows clinicians to have an additional resource with which to refer their cancer survivor patients [15]. Third, researchers may leverage the programs for potential community-based collaborations or implementation of future study protocols among cancer survivors. The inclusion of post-secondary courses was considered important because qualified exercise professionals play a central role in program delivery [15, 25]. Furthermore, training and education in the exercise oncology field are important for developing and leading appropriate and safe cancer-specific physical activity programs [26]. Thus, including post-secondary courses in the search was prudent.

While the COREN website was developed with national resources in mind, the current systematic scoping study methods were developed within the context of Ontario, Canada. The rationale was that the methods could be established within one province first and later adapted and extended to other geographical regions. Moreover, a large portion of cancer survivors reside in Ontario, which in turn has implications for the number of cancer-specific physical activity and post-secondary opportunities available [27].

The purpose of the current investigation is to describe the application of the systematic scoping study methods in the context of cancer-specific physical activity programs and post-secondary courses in Ontario, Canada. The objective of the investigation is to provide a methodological adaptation for future searches of “real-world” opportunities in health-related contexts. Key findings of the search are highlighted, and reflections on the search methods, findings, and future directions are discussed.

## Methods

While the current investigation was not a systematic scoping review, reporting followed the Preferred Reporting Items for Systematic reviews and Meta-Analyses extension for Scoping Reviews (PRISMA-ScR [28]) to provide transparent reporting of the methods used. Refer to Additional file 1 for the PRISMA-ScR checklist. Prior to conducting searches, ethical approval was obtained from the host institution’s Health Sciences Research Ethics Board. Searches for cancer-specific physical activity programs and post-secondary courses were conducted from January 2018 to June 2019. Search results were referred to as “records.” Records were operationalized as any website, program, and course documentation (e.g., description or syllabus) with potentially relevant information. The current investigation was guided by the previous framework for systematic scoping reviews [29, 30], which included the following steps: (a) identify the research aim (refer to purpose and objective); (b) identify relevant search records; (c) select records; (d) chart the data; (e) collate, summarize, and report results; and (f) consultation. In contrast to previous research, consultation occurred simultaneously with identifying relevant search records. The protocol has not been registered.

### Cancer-specific physical activity programs

#### Eligibility criteria

Refer to Table 1 for inclusion and exclusion criteria for cancer-specific physical activity programs.

**Table 1:** Inclusion and exclusion criteria for cancer-specific physical activity program and post-secondary course search

Inclusion criteria	Exclusion criteria
Physical activity program search	
Appropriate for adults (≥ 18 years)	Specifically targets children (≤ 17 years)
Intended for adults diagnosed with cancer Appropriate for adults in an older adult population	Specifically targets persons in the general population or other chronic conditions that are not cancer
Offered in the community	Delivered in laboratory, assisted living, or similar settings
Delivered in Ontario	Delivered outside of Ontario
Physical activity programs were described as a group or individual sessions Programs included structured exercise, sport, lighter intensity exercise (e.g., yoga, Tai Chi)	Described as physical activity guidelines or recommendations, only Described informational and/or training resources
Offered a regularly scheduled session (e.g., weekly, daily, bi-weekly)	Sessions were no longer running on a regular schedule
Post-secondary course search	
Currently offered at a college or university in Ontario	Specifically targets elementary and/or high school curriculum and/or located in another province
Course content included at least one lecture on cancer and exercise/physical activity as verified by syllabus and/or course instructor	Did not contain any course content on cancer and exercise

#### Information sources and search strategies

Consistent with Godin and colleagues [18], a search plan was developed. The terms used for the physical activity program search are as follows: (a) physical activity (or exercise), (b) community (or program), (c) cancer survivor, and (d) older adult. The search plan was quite broad in order to capture any physical activity program that could be appropriate for cancer survivors, including those developed for clinical populations such as older adults. Information sources included all websites which met eligibility criteria.

Searches were independently completed by a co-author (KK) and an undergraduate research assistant (HS) and initially reviewed by another undergraduate research assistant (NG). All independent searches occurred approximately 3 to 4 months apart. All undergraduate research assistants were trained on study-specific protocols by one of the co-authors (i.e., AF, KK, and JT).

##### Google Advanced Search

The search terms were entered into *any of these words* field. To increase the likelihood that websites from Ontario, Canada, were found, *region* field was selected for Canada as specific geographic regions, including provinces, were not available. All remaining fields were left at the default setting or remained blank. Given that a Google Advanced Search would likely yield a high number of search results and that most relevant searches would be displayed first, an a priori decision was made to limit the data extraction to the first 20 pages (i.e., 200 results) to capture the most relevant records [17].

##### Targeted website search

The search was conducted to identify relevant cancer-specific websites using a Google search. Search terms included (“cancer survivor” AND “community” AND “service” AND/OR “program”) to find potential websites. A filter was applied to only include Canadian websites. Similar to the Google Advanced Search, only the first 20 pages were searched to capture the most relevant records. Two relevant websites were identified including the Canadian Cancer Society and Cancer Care Ontario. The Canadian Cancer Society Community Service Locator (https://csl.cancer.ca/en) was hand searched to find potentially relevant physical activity programs through the custom search function using the search terms “exercise rehabilitation” as suggested on the website. The website had a map feature that was used to narrow the geographic region to Ontario. The Cancer Care Ontario website (https://www.cancercareontario.ca/en) was also hand searched using the website’s custom search using the terms “exercise program” or “physical activity program.”

##### Consultation with content experts

A content expert was defined as an individual who worked directly with cancer survivors in a community-based or clinical physical activity, exercise, or rehabilitative setting with knowledge of available physical activity programs for cancer survivors. Content experts were identified through a knowledge transfer event for physical activity and cancer survivorship [31]. Interested individuals were contacted via email or phone regarding participation. Content experts who agreed to participate provided written informed consent. Content experts were asked to identify any potentially relevant physical activity programs and/or forward the request to a colleague who would be willing to assist. A reminder email was sent approximately 7 days following initial contact if there was no response. Content experts were contacted a maximum of three times by either phone or email. Forty-two content experts were contacted, and of those, *n* = 17 provided information. Content experts who provided information received a CA$10 gift card compensation.

#### Selection of information sources

##### Identification through title screens

Potentially relevant records from the first 20 pages of the Google Advanced Search and targeted website searches underwent a title screen. All physical activity programs identified by content experts were included. All potentially relevant records from the three search strategies were then copied and pasted into an Excel spreadsheet. Undergraduate research assistants independently removed duplicates using the find duplicates function in Excel and visually searching the database. After removing duplicates, each URL was clicked to determine if the link still led to an operational website.

##### Record selection through full review and verification

Each operational website URL underwent a full review. The full review included a hand-search of the entire website for relevant information about a cancer-specific physical activity program as per eligibility criteria (refer to Table 1) and verification that the program was still active. Two independent reviewers (AF and KK) conducted the full review and disputes over programs to include were settled through discussion. All eligible website URLs that identified a cancer-specific physical activity program were recorded in a separate Excel spreadsheet. To verify that programs were still active, an undergraduate research assistant contacted all eligible physical activity programs over the phone. Programs with disconnected numbers were excluded. The undergraduate research assistant phoned the programs up to three times over the course of 14 days and left voicemails explaining the purpose of the call. If a voicemail could not be left, an email was sent explaining the purpose of the call. If no response was rendered, it was assumed the program was no longer active and it was excluded.

##### Data charting process and data items

A data charting form was developed based on information that would be relevant for cancer survivors on the COREN website and previous research [9, 17]. Data charting was conducted iteratively and independently by two undergraduate research assistants. Subsequently, data were reviewed for completeness by the lead author. Data items included program name, cancer site, program description, location (city), phone number, and/or URL. Where physical activity programs had multiple locations, the record was counted once, and all location data were extracted. Locations were categorized into urban and rural regions based on Ontario census data [27]. The programs were categorized into either structured exercise or recreational programs following extraction. Structured exercise programs included defined exercise prescription with the goal of meeting exercise guidelines or structured rehabilitation programming (e.g., physical therapy). Recreational programs did not include a defined exercise prescription, but rather included physical activities such as yoga, Qi Gong, water aerobics, and sports such as dragon boat.

### Post-secondary courses

#### Eligibility criteria

Refer to Table 1 for inclusion and exclusion criteria for post-secondary courses.

#### Information sources and search strategies

The search terms used were (a) cancer, (b) physical activity (or exercise), (c) clinical population (or special population), and (d) chronic. The search terms clinical population, special population, and chronic were included because cancer-related material could be located within course descriptions and/or syllabi. Information sources were post-secondary institutional websites, course descriptions, and course syllabi or similar materials.

Post-secondary course searches were independently completed by two undergraduate research assistants (SS, DB) and initially reviewed by one undergraduate research assistant (NB). All independent searches occurred approximately 3 to 4 months apart. The lead author reviewed potentially relevant records from all independent searches to identify discrepancies between the searches.

##### Targeted website search

Post-secondary institutions, including colleges and universities, were identified using a targeted website search through the centralized government website (https://www.ontario.ca/page/go-college-or-university-ontario#section-1). The centralized government website has two separate webpages listing all 24 colleges and 22 universities in Ontario. A link to each institution was then clicked and the online course catalogue or calendar for each institution was located. The search terms were entered into each institution-specific course catalog separated by commas (cancer, physical activity, exercise, clinical population, special population, chronic). Similar to the Google Advanced Search, the first 20 pages of results were searched to capture any relevant records per institutional website.

#### Selection of information sources

##### Identification through title screen

The same method for title screening cancer-specific physical activity programs was followed.

##### Record selection through full review and verification

The full review included confirming eligibility criteria (refer to Table 1) by searching the course description and course verification. To complete the verification, course descriptions and syllabi were scanned for relevant content on physical activity and cancer. Where the syllabus was not available, the named course instructor was contacted by phone or email. The course instructor was asked to verify whether cancer and physical activity were discussed in the course, the extent to which the target topic was discussed or the number of lectures, and if the course would be offered in the 2019–2020 academic year. Where the course instructor could not be contacted, the registrar’s office of the institution was contacted by phone or email. Course instructors and/or registrar officials were contacted up to three times with approximately 4 to 7 days in between each contact. If there was no response after the third contact, it was assumed the course did not contain cancer-related material and was excluded.

##### Data charting process and data items

The same data charting process for cancer-specific physical activity programs was followed. The data items included institution name, department, course title, course code, description, instructor name, course length (i.e., semester, full year, not indicated), and level (i.e., undergraduate, graduate, or graduate [professional]). Eligible post-secondary courses were categorized as theoretical courses, which are courses that did not have a practical or laboratory component (i.e., developing and delivering exercise prescriptions for cancer survivors), or practical courses, which did include the aforementioned components.

## Results

The cancer-specific physical activity programs and post-secondary opportunities have been posted on the COREN website (http://www.icanbeactive.com/).

### Cancer-specific physical activity programs

**Fig. 1:**
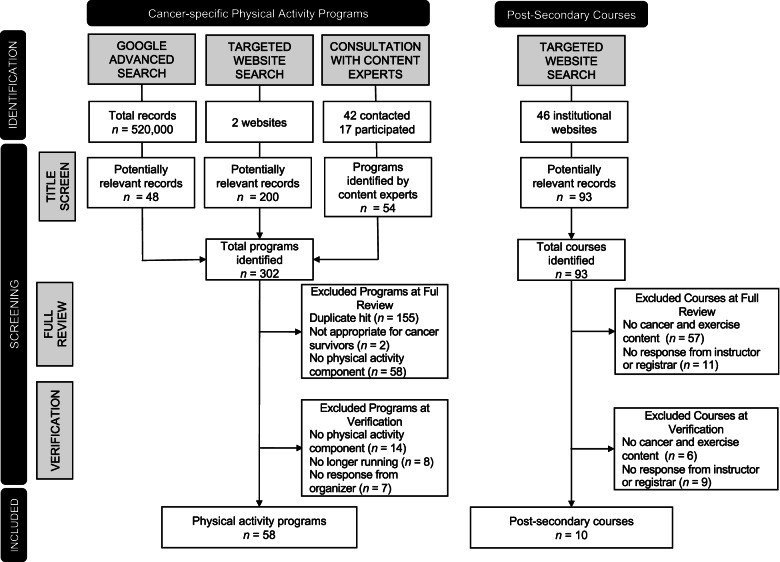
Flow diagram of the cancer-specific physical activity program and post-secondary course selection for cancer survivors

Across the three search strategies, *n* = 302 potentially relevant records were screened and verified. Refer to Fig. 1 for a flow chart of the findings. After the full review and verification procedures, *N* = 58 (*n* = 20 structured exercise and *n* = 38 recreational) programs were included on the COREN website. Refer to Additional file 2: Table 2 for programs. All physical activity programs appeared in at least two search strategies. Physical activity programs were offered mostly in urban settings (*n* = 45; 77.6%) followed by rural settings (*n* = 9; 15.5%). Within the rural setting, only *n* = 4 (6.9%) programs were offered in Northern Ontario, which is a less densely populated and remote region of the province.

### Post-secondary courses

A total of *n* = 93 potentially relevant records were identified using the targeted website search and all were screened through full review and verification procedures. Ten post-secondary courses were included on the COREN website. Refer to Additional file 2: Table 3 for post-secondary courses. Post-secondary courses were offered at *n* = 8 unique institutions that were mostly universities (*n* = 5, 62.5%). One institution offered the most courses (*n* = 3; 30.0%) with the remaining institutions offering one course each. Courses were offered at the undergraduate level (*n* = 4, 40.0%), followed by graduate- or professional graduate-level (*n* = 3) and college-level (*n* = 3). Courses were mostly one semester in duration (*n* = 6; 60.0%). Based on the syllabi, course descriptions, and discussions with course instructors and/or registrar officials, most (*n* = 70; 70.0%) of courses were theoretical in pedagogical nature and focused on biobehavioral barriers, facilitators, and mechanisms of physical activity among individuals diagnosed with cancer.

## Discussion

This paper described the application of systematic scoping study methods to identify cancer-specific physical activity and post-secondary courses in Ontario, Canada. The search method was adapted from recommendations for the conduct of systematic scoping reviews of the gray literature [18] and searches for “real-world” initiatives [17]. Three search strategies were used: Google Advanced Search, targeted website searches, and consultations with content experts. Fifty-eight physical activity programs and 10 post-secondary courses were identified that met the eligibility criteria. Reflections on applying systematic scoping study methods and the findings are discussed below.

### Reflections on applying systematic scoping study methods

Three search strategies were used to find cancer-specific physical activity programs and post-secondary courses. The most successful search strategy for cancer-specific physical activity programs was the targeted website search followed by Google Advanced Search and content expert consultations. These findings are consistent with previously developed search strategies for community-based physical activity programming for persons with disability [17]. Similar to D’Urzo and colleagues, physical activity programs were identified across multiple strategies, which suggests feasibility for integrating multiple strategies into a comprehensive search method [17]. One search strategy was used to search for post-secondary courses with cancer-related content. While the search method likely yielded most of the current post-secondary courses, it is possible that some courses may have been missed. Other types of post-secondary institutions, such as specialist or private institutions which focus on a particular skill or trade (e.g., respiratory therapy), were not searched; doing so may have also yielded unique results.

Lack of reporting standards for “real-world” opportunities has an impact on the reliability of potentially relevant records [17], which may introduce bias into the search methods [18]. In the current investigation, there was an abundance of potentially relevant records using Google Advanced Search that matched the search terms, yet only 48 potentially relevant records were identified using this search strategy. For the post-secondary course search, course titles and descriptions had ambiguous information. To mitigate potential bias, a search plan was implemented and including full review and verification procedures. Heeding guidance from Godin and colleagues [18], a search plan was implemented to reduce the risk of introducing bias and offer guidance and structure to the search, which promotes transparency. Additionally, cancer-specific physical activity programs were verified by contacting program organizers and post-secondary courses were verified by examining course syllabi and contacting the course instructors and registrar officials directly. Verification excluded additional records (refer to Fig. 1) and this was a critical step during the screening procedure to identify relevant and current cancer-specific physical activity programs and post-secondary courses.

The systematic scoping search methods used in the current investigation represent a “snapshot” in time. The ever-changing nature of URLs and lack of Internet archiving leads to potentially relevant records changing or disappearing over time [20]. The change or loss of information decreases the reproducibility of the search strategies and makes it difficult to update the systematic scoping study method [18, 20, 32, 33]. In an effort to rapidly accumulate information and maintain rigor of a systematic scoping study method, a search including “grey data”—user-generated data that is Web-based, for example social media and blog posts [20]—may be utilized in future investigations. For example, researchers could post requests for relevant information on Twitter and ask followers to re-tweet. The mention function could be used to identify additional physical activity programs by including accounts of relevant organizations [20].

### Reflections on search findings

The cancer-specific physical activity programs included (*n* = 58) were located in mostly urban or suburban areas. Specifically, only four programs were found in the northern, remote region of the province, which suggests that this area is underserved. Cancer survivors living in rural settings have unique physical activity preferences [34] and implementation strategies for community-based programs may need to address barriers such as cost, necessary expertise, and lack of awareness [35]. Furthermore, the Internet and technology-supported strategies should also be leveraged as broadband access is increasing in rural areas [35].

Few relevant post-secondary courses (*n* = 10) are offered in Ontario. As a result, post-secondary students aspiring to become exercise professionals may not have exposure to relevant course content. Exposure to content is relevant for building foundational knowledge in the exercise oncology field [26]. Most post-secondary courses were theoretical, which is valuable for understanding the effects of physical activity on cancer survivorship. However, the inclusion of experiential learning or hands-on approaches in post-secondary education can be equally as valuable for individuals wishing to become qualified exercise professionals in exercise oncology by improving skill proficiency and knowledge [26].

### Strengths

Despite the challenges and limitations described above, there are some strengths that can be gleaned from the current investigation. First, the findings suggest that the systematic scoping study methods can be applied in various contexts, thus contributing to its versatility [18]. Second, a priori definitions of the content needed for the knowledge tool (COREN website), target audience (researchers, clinicians, and survivors), and outcomes (physical activity programs and post-secondary courses) helped to narrow down the information that was ultimately searched [29, 36]. The definitions enhanced the efficiency of searches, which has been a limitation of previous investigations using systematic scoping study methods (e.g., [20]). Third, the inclusion of various types of physical activity modalities from structured exercise programs, Qi Gong, and sport led to a comprehensive list of programs that have the potential to suit survivors’ multiple abilities and interests across the cancer survivorship trajectory. Additionally, these programs represent potential for community-based collaborations for researchers as well as physical activity recommendations for clinicians to provide their patients.

### Future directions

Future research is encouraged to use the presented methods as a guide for searches of “real world” opportunities in various health contexts, including online delivered cancer-specific physical activity programs and other education opportunities. Applying the current search strategies to online opportunities would be novel given that many organizations and institutions needed to pivot programming and course instruction to an online setting during the COVID-19 pandemic. Future research is encouraged to apply the current systematic scoping study methods to education opportunities outside of formal post-secondary settings as part of the search. For example, applying the methods to qualified exercise professional training certification programs may be valuable. Training certification program course content is focused on cancer and exercise for professional settings and may inform the versatility of the current methods [15, 25]. Finally, future research is encouraged to consider quality assessments. Similar to Arksey and O’Malley [30], the current investigation did not directly relate to quality assessment. However, it is acknowledged that a lack of quality assessments may limit the uptake of findings into practice [37] and there are challenges assessing the quality of a variety of records [30].

## Conclusions

In conclusion, using systematic scoping study methods in “real world” opportunities is a valuable source of information for pragmatic investigations with defined content needed for knowledge tools. Challenges were experienced due to lack of standard reporting among physical activity programs and post-secondary courses, bias of potentially relevant records, and changing nature of resulting records. The current investigation demonstrated that systematic scoping study methods can be applied to cancer-specific physical activity programs and post-secondary courses in the context of cancer survivorship in Ontario yielding robust results. The search method can be further adapted and updated in future health-related knowledge syntheses in other contexts.

## Supplementary Information

**Additional file 1.** Preferred Reporting Items for Systematic reviews and Meta-Analyses extension for Scoping Reviews (PRISMA-ScR) Checklist.

**Additional file 2. **Complete search results for cancer-specific physical activity programs and post-secondary courses.

## Data Availability

The datasets used and/or analyzed during the current study are available from the corresponding author on reasonable request.
