# Supplementary material for: Adapting systematic scoping study methods to identify cancer-specific physical activity opportunities in Ontario, Canada
Source: Syst Rev. 2022 Jan 18;11:13. doi: 10.1186/s13643-022-01886-8 (PMC8765105; doi:10.1186/s13643-022-01886-8)
Supplement: Supplementary file 2 — Additional file 2. Complete search results for cancer-specific physical activity programs and post-secondary courses. [file 13643_2022_1886_MOESM2_ESM.docx]

Additional File 2 - Table 2

*Summary of Physical Activity Programs for Cancer Survivors in Ontario (N = 58)*

| Program | Cancer Site | Brief Description | Location (City) | URL or Telephone Number* |
| --- | --- | --- | --- | --- |
| Structured exercise programs (*n* = 20) | | | | |
| Encore YWCA | Women with cancer | The YWCA offers ENCORE, an 8-week floor and pool exercise program (one 2-hour session/week) to people who have had breast cancer at any time in their lives. | Bracebridge, Guelph,  Hamilton | http://www.ywcamuskoka.com/womens-programs/ywcaencore  http://www.guelphy.org/y-in-the-community/encore/ |
| CanWell at YMCA Les Charters | All types | The Les Charters YMCA in Hamilton offers a 12-week supervised exercise program for individuals diagnosed with cancer at any stage of cancer treatment. | Hamilton | http://www.hhsc.ca/body.cfm?id=1962 |
| MacWarriors at McMaster University | All types | Physiotherapists and health professionals have the expertise to ensure all our clients reap the benefits of cancer rehab and exercise (community-based). | Hamilton | https://pace.mcmaster.ca/programs/mac-warriors-cancer-rehabilitation |
| CAN-soar: Cancer Rehabilitation | All types | Cancer Rehabilitation: CAN-soar is a 10-week exercise and physiotherapy program. It includes an individualized exercise program to suit your specific needs with manual therapy provided by a physiotherapist. It is a supervised circuit-training program. It is okay to have fatigue, pain, weakness, swelling, dressings, cording, fibrosis, expanders, implants: CAN-soar will work through these with you. | Hamilton | http://www.physiocareservices.com |
| The Core Family Health Centre -- Skye Physiotherapy & Pilates | All types | A variety of Pilates Classes are offered by Physiotherapists. Classes are small to ensure individualized attention for each participant. A variety of resistance and stability equipment is utilized. | London | http://www.skyehealth.ca/Classes/pilates-classes-in-London-Ontario/a~2381--c~331184/article.html |
| Cancer Transitions at Stronach Regional Cancer Centre | All types | 6-week program to help cancer survivors transition from treatment to regular lives. | Newmarket | https://southlake.ca/patient-care-program/cancer/#nav-programservice_clinics |
| Cancer Rehabilitation at Haley Rehab | All types | Private physiotherapy clinic specialized in cancer, breast cancer, lymphedema, and deep vein thrombosis. No physician referral necessary. Our goal is for you to get back to doing the things you love, with the best quality of life possible. | Ottawa | www.haleyrehab.ca |
| Exercise in Oncology at Kinnect to Wellness | All types | Rehabilitation: Moderate exercise classes help people to improve quality of life, gain strength, and address fatigue that may be brought on from cancer treatments. | Sudbury | http://www.kinnecttowellness.ca |
| WE-Can at Canadian Games Complex | All types | 10-week wellness and exercise program for individuals living with cancer. | Thunder Bay | http://www.tbrhsc.net/wp-content/uploads/2015/12/WE-Can_brochure_2015.pdf |
| Wellspring Exercise Program (additional programming including yoga) | All types | Wellspring's cancer exercise program was specifically developed for the needs of cancer patients by a registered physiotherapist and pioneer in cancer rehab Ontario. Patients receive an individual assessment, personalized exercise program, and opportunity to exercise with other patients, and instruction on self-managed exercise both during and following the program. | Toronto (Downtown and Westerkirk House at Sunnybrook), Halton Peel (Birmingham Gilgan House) | https://wellspring.ca/centre-of-innovation/cancer-exercise/ |
| Gilda's Club (several programs) | All types | The following group exercise programs are available: Men's Yoga, Yoga, Core Strength Class, Electric Chair Yoga, Zumba, Creative Movement with the National Ballet School, Pilates. | Toronto | https://gildasclubtoronto.org |
| The Health, Exercise, Active Living and Therapeutic Lifestyle (HEALTH) Program at University Health Network | Breast | A 6 month program offering a unique blend of education, exercise and peer support. The program provides: Individually prescribed aerobic (cardio) and resistance training exercise, education about topics related to exercise, breast cancer and heart health, a supportive place for women who are at different stages of breast cancer treatment. | Toronto | http://www.uhn.ca/TorontoRehab/PatientsFamilies/Clinics_Tests/HEALTh |
| Cancer Rehabilitation and Survivorship Program: Cancer Rehabilitation and Exercise (CaRE) Program at University Health Network | All types | Comprehensively assess the survivorship and rehabilitation needs of individuals with cancer, teach participants about exercise and how to perform it safely, test fitness levels and help understand what it means, 8 weeks of group-based exercises, educational classes related to cancer survivorship issues. | Toronto | http://www.uhn.ca/PrincessMargaret/PatientsFamilies/Clinics_Tests/Cancer_Rehab_Survivorship/Pages/care_program.aspx |
| Toronto Rehab Ambulatory musculoskeletal and oncology programs | All types | Assessment by a team of health care professionals to address issues in mobility and overall function caused by cancer diagnosis and or treatment | Toronto | https://www.uhn.ca/TorontoRehab/PatientsFamilies/Clinics_Tests/Musculoskeletal_Rehabilitation |
| Cancer Rehabilitation at the Orthopedic Therapy Clinic | All types | Registered Kinesiology & Therapeutic Fitness helps individuals living with cancer minimize disability and achieve the highest possible return to function. They provide an individualized approach to rehabilitation and teach you the skills you need to help restore your independence. | Toronto | http://www.orthophysio.com |
| Oncology Rehabilitation Program at Orthopedic Therapy Clinic | All types | For patients who have undergone, or are planning for, cancer surgery or cancer treatment. This short-stay, active rehabilitation program helps patients increase their strength, mobility and independence for a smooth return to the community and their lives. | Toronto | http://sunnybrook.ca/content/?page=SJR_patvis_prog_onc |
| Cancer Smart Rehab at MedFit Rehab | All types | A holistic approach to ensure we progressively engage the mind, body, and spirit as we strive to improve client strength, ROM, endurance and overall quality of life. (Team: Exercise rehab specialists, medical exercise specialists, kinesiologists and gerontologists). | Toronto | http://medfitrehab.com |
| LifeMark Physiotherapy/  Centric Health Cancer (C.A.R.E ) | All types | A private physiotherapy clinic that offers cancer rehabilitation at designated facilities across the country. | Various  (refer to website for specific locations) | www.lifemarkphysio.ca/services/cancer-care |
| Living Fit, Well Fit, Well Fit Grad Program | All types | The UW WELL-FIT 12-week program focuses on restoring and improving the physiological well-being of individuals on chemotherapy, radiation or hormonal therapy treatment. | Waterloo | https://uwaterloo.ca/uw-fitness/uw-well-fit/uw-well-fit-12-week-program |
| Renew at Windsor Squash and Fitness | All types | RENEW Back to fitness program - Designed for cancer survivors and delived by certified professionals from Windsor Squash & Fitness in collaboration with the Windsor Regional Hospital Cancer program (on-site). | Windsor | http://windsorite.ca/events/renew-back-to-fitness-program-61/ |
| Recreational programs (*n* = 38) | | | | |
| Hope House | All types | Certified professionals volunteer their time to provide services that promote health & wellbeing. Programs include yoga, meditation, walking, 1:1 modalities such as reflexology, Reiki, healing touch, etc. | Aurora,  Richmond Hill | https://www.hopehousehospice.com/page3 |
| Restorative Yoga (Home Studio) by Kathleen Gorden | All types | In her home studio, Kathleen Gorden instructs Restorative yoga as a healing and gentle form of exercise. Private classes offered. | Brantford | http://www.turningpointnutrition.ca/cancer-care-in-brantford-ontario |
| Evergreen Yoga- Yoga for Cancer Warriors | All types | Yoga for Cancer Warriors is for those newly diagnosed, in treatment, post treatment, gathering strength, thriving or for those loved ones supporting a Warrior. The sessions are held on a one-on-one basis either in our peaceful Caledon studio or in the comfort and convenience of the client's home. They are tailored to the individual's needs, objectives and goals. | Caledon | http://www.evergreenyoga.ca/yoga-for-cancer-warriors.html |
| Living Yoga & Health | All types | Qualified instructors offer classes for prenatal, toddlers, children, teens, adults, and those over 50. Programs offered include gentle yoga, restorative yoga, therapeutic yoga, yoga meditation, and many more. | Guelph | https://livingyoga.ca/class-descriptions-2-3/ |
| Breath Rejuvenation Studio | All types | Breathe Rejuvenation Studio is downtown Guelph's natural organic aesthetic spa and yoga studio for both men and women. Breath is ecologically aware, combining organic ingredients, and local and fair-trade practices into a professional variety of quality wellness services. | Guelph | https://www.visitguelphwellington.ca/listing/2177 |
| BreastStrokes Dragon Boat Team | Breast | Dragon Boat | Guelph | http:/www.breaststrokes.org |
| Aqua Motion, YMCA Hamilton | Breast | Specifically designed for women who have had breast cancer at any time in their lives. Gentle exercises that use buoyancy and resistance training to improve range of motion, endurance and reduce lymphoedema. | Hamilton | http://ywcahamilton.org/what-we-do/health-wellness##AquaMotion |
| Pilates Beyond Breast Cacner, YMCA Hamilton | Breast | Pilates Beyond Breast Cancer is designed to build stamina, improve posture, increase shoulder range of motion, regain function and release neck tension with the help of a certified Pilates instructor. | Hamilton | http://ywcahamilton.org/what-we-do/health-wellness##Pilates |
| Knot A Breast | All types | Dragon Boat | Hamilton | http://www.knotabreast.com |
| Wellwood Hamilton (Juravinski House)- Qi Gong | All types | No previous experience is necessary to begin this program. Comfortable clothing is recommended. Tai Chi movements are very graceful and flow "like water and clouds." Its many health benefits include building strength in joints and balancing the body. | Hamilton | http://www.wellwood.on.ca/index.php/our-programs/12-qi-gong-for-individuals-affected-by-cancer |
| Into the Flow Yoga and Wellness | All types | A specialized yoga methodology that is tailored to address the specific physical and emotional needs left by cancer & its treatments. Community classes are free at the YMCA. | Kingston | https://intotheflow.ca |
| Breast Cancer Action Kingston (BCAK) – Lymphatic Yoga | Breast | All classes are held in the fitness room at BCAK and are open to breast cancer patients with a current paid up BCAK membership. All participants require the approval of their physician to participate. | Kingston | http://bcakingston.ca/programs-services/fitness-classes/ |
| Breast Cancer Action Kingston – Urban Poling | Breast | All classes are held in the fitness room at BCAK and are open to breast cancer patients with a current paid up BCAK membership. All participants require the approval of their physician to participate. | Kingston | http://bcakingston.ca/fitness-classes/ |
| Rowbust Dragon Boat | Breast | Dragon Boat | London | https://www.rowbustdragonboat.com/ |
| After Breast Cancer Diagnosis Aquafit | All types | Relax with focused breathing while increasing strength and range of motion. Gentle movement in the water will assist in circulation to reduce lymphatic swelling held in the warm water therapeutic pool. | Mississauga | https://www.mississaugahaltonhealthline.ca/displayService.aspx?id=164719 |
| Aquafitness Healing Waters, François Dupuis Recreation Centre | All types | Post-rehabilitation program in the water provides support and relieves weight-bearing pressure on joints. May improve functional mobility for persons who have experienced prolonged joint immobilization due to chronic or acute health conditions. | Orleans,  Ottawa | http://join.ottawa.ca/fac/247/winter/all/act/12/6016/ |
| Hearth Place Cancer Support Center | All types | Providing community support for people diagnosed with cancer and their families through individual and group support, information, a resource centre, wellness programs and an ongoing lecture and discussion series.  Several wellness programs offered free of charge. A doctor’s note is required. | Oshawa | http://hearthplace.org/wellness-programs/ |
| Aquafitness for Cancer Survivors, Dovercourt  Recreation | All types | Supervised program geared toward those newly diagnosed, undergoing treatment, or in recovery. | Ottawa | https://www.dovercourt.org/ |
| Ottawa Integrative Cancer Centre – Gentle Yoga | All types | Supervised program open to people at any stage of the cancer journey; from diagnosis to treatment to those post treatment, caregivers, and staff at the OICC. | Ottawa | http://www.oicc.ca/en/programs-event/programs/gentle-yoga |
| Breast Cancer Action Ottawa | All types | The emphasis is on getting fit, doing activities safely especially in view of the surgeries we have undergone for our breast cancers, and having FUN. We offer yoga, stretch and strength (which aims to be about half aerobics and half stretching and resistance training, belly dancing, Tai Chi, and now pole walking as an intermittent offering. | Ottawa | http://bcaott.ca/health-and-wellness-program/ |
| Ottawa Hospital Cancer Centre- Yoga Thrive | All types | Support and rehabilitation to patients and their families during treatments and follow-up. The team includes: social workers, dietitians, physiotherapists, speech and language pathologists, psychologists and psychiatrists. Covered by Ontario Health Insurance Plan. | Ottawa | https://easternontario.cioc.ca/record/OCR2728 |
| Surround Circle Yoga- Restorative Yoga | All types | Restorative Yoga offers tremendous tension release and deep physical, emotional and mental rest for those struggling with everyday stress and fatigue, limited mobility, or living with chronic conditions. 12-week series $210 or drop in $20. | Ottawa | http://surroundcircleyoga.com/classes/ |
| Make Waves – Aquafit, offered at the Ray Friel Centre and the Orleans Recreation Complex | All types | No description available, please call for information and location. | Ottawa | Ray Friel Centre - 613-830-2747;  Orleans Recreation Complex - 613-824-0819 |
| AquaCan | All types | Supervised, 45-minute, seasonal classes. Classes run twice a week, members and non-members welcome. | Ottawa | (613) 798-9818 |
| Yoga Therapeutics - Yoga for Lymphatic Health | All types | We will explore how yoga breath; specific yoga postures and relaxation can aid in the irrigation of lymph throughout the body. You will learn the role the skin plays and how to stimulate lymphatic flow via the skin. | Peterborough | http://www.yogatherapeuticspeterborough.ca/services-for-yoga-therapy.html#lymphatic |
| Taoist Tai Chi Society - Health Recovery Classes | All types | Ongoing supervised classes and programs. Participants are encouraged to come to as many classes as they would like. | Sault Ste. Marie | https://www.taoist.org/locations/sault-ste-marie/ |
| Cancer Support and Resource Program – Tai Chi Classes | All types | Support group for women with any type of cancer and their caregivers if they wish to bring them. Open to all women dealing with ANY type of cancer or long-term effects of treatment, as well as survivors. Facilitated by a peer support volunteer with training in peer support and group facilitation. | Simcoe | http://www.csrp.ca/support-groups-agencies/ |
| Yoga and Wellness For Life – Yoga Therapy | All types | Yoga therapy applies the entire science of yoga to effect health and healing on an individual basis. Services are offered by Certified Yoga Therapists. After an initial intake consultation, you will customize a program that combines at-home work with one-on-one, in-person appointments. Initial consultation $110 and $60 per yoga therapy session (60 minutes). | Smith Falls | https://www.smithsfallsyoga.com/yoga-therapy |
| Dragons of Hope | Breast | Dragon Boat | Thunder Bay | https://www.facebook.com/dragonsofhope/ |
| Fitzone Plus- Pilates with Samantha | All types | Personal Pilates program supervised by Samantha in a group setting or home-based setting. | Toronto | https://www.pilateswithsamantha.com/ |
| ELLICSR- Taoist Tai Chi Arts | All types | This class includes gentle movement, breathing exercises and meditation. Classes are held once a month and registration is required. | Toronto | https://www.ellicsr.ca/en/classes_events/classes/Pages/taoist_tai_chi_arts.aspx |
| Healthy Steps ELLICSR | All types | Fun stretches and dance moves set to music in a healing exercise program designed to help you thrive. Registration required. | Toronto | https://www.ellicsr.ca/en/classes_events/classes/Pages/healthy_steps.aspx |
| Dragons Abreast | Breast | Dragon Boat | Toronto | http://www.dragonsabreast.ca/ |
| Pilates with Samantha at Fitzone-Plus | All types | She provides a Pilates-based exercise program designed to help with the following: restore range of motion in the affected arm, increase overall strength and flexibility, improve posture and alignment, restore breathing and lung capacity, enhance self-image and wellbeing. | Toronto | http://www.pilateswithsamantha.com |
| Running Room Survivor Training Programs- Walk/Run | All types | FREE training programs are 10 weeks in duration and are open to walkers and runners who are breast cancer survivors. The 10 week "For Women Only" Learn to Run training program is structured entirely around women's needs and will give participants a chance to network with other women who have faced similar challenges with their health. | Various | https://www.events.runningroom.com/training/?sid=622&id=5172 |
| HopeSpring Cancer Support Centre | All types | These supervised programs teach proven techniques that reduce feelings of stress, calm emotions, and help you feel more in control of your experience, and how you think and feel. | Waterloo, Kitchener and Cambridge | https://www.hopespring.ca/programs/relaxation__movement_programs/ |
| YogaBliss | All types | All classes offer a compassionate yoga style that invite you to create the practice that is appropriate for your body and your energy level in each moment. The YogaBliss studio provides a sanctuary to wander through the yogic journey uncovering the layers that bring inner peace and contentment. | Whitby | https://yogabliss.me/class-description/ |
| Windsor Hospice Wellness Programs- Tai Chi | All types | This program consists of a simplified form of tai chi with emphasis on slow turning and stretching along with a form of Qi Gong known as Ba Da Jin. This is a great way to relax and improve your health. | Windsor | http://www.thehospice.ca/article-18/patient-family-programs-wellness-centre |

*Note.* *Current as of June 2019.

Additional File 2 - Table 3

*Summary of Post-Secondary Courses with Cancer and Exercise Content (N = 10)*

| Institution and Department | Course Title and Code | Brief Course description | Prerequisite Requirements | Course Instructor | Course Length | Level |
| --- | --- | --- | --- | --- | --- | --- |
| University courses (*n* = 7) | | | | | | |
| University of Toronto  Faculty of Kinesiology and Physical Education | Exercise & Cancer Survivorship  KPE 426H | This course will examine the role of exercise and physical activity in cancer survivorship from a physiological, functional, and psychosocial perspective. The effects of exercise and physical activity on cancer will be studied across the disease continuum, including its role in cancer prevention, disease and treatment related symptom management, post-treatment late-effects, and palliative care. Exercise guidelines, required adaptations, appropriate fitness and outcome assessments for cancer survivors will be discussed. | KPE 220H Psychosocial Development  OR  PHE 101Y  OR  PSY 210H | Santa Mina | Semester | Undergraduate |
| University of Toronto  Faculty of Kinesiology and Physical Education | Special Topics in Exercise Oncology  EXS5538H | This course is designed to provide an overview of the role of physical activity in cancer control. Evidence for the effectiveness of physical activity in coping with treatments, recovery after treatments, and survivorship will be examined. The objectives of the course are to: (1) obtain a basic understanding of cancer including its epidemiology, treatments, and side effects, (2) gain a comprehensive understanding about the role of exercise for cancer survivors during and after treatment, (3) identify the key outcomes and determinants of physical activity, and (4) understand the effects of sedentary behavior for cancer prevention and survivorship. |  | Trinh | Semester | Graduate |
| University of Toronto  Kinesiology and Physical Education- Master of Professional Kinesiology (MPK) | Placement: Chronic Disease and Mental Health  MPK2010H/  MPK4011H | MPK students will contribute to the delivery of the U of T Secondary Prevention and Rehabilitation Kinesiology (S.P.A.R.K.) program with the aim of promoting long-term health through exercise prescription and physical activity participation. In the S.P.A.R.K. program, MPK students are paired one-on-one with an adult with a previous history of cancer or cardiovascular disease for the purpose of assessing exercise needs and  developing and delivering an exercise plan. | Registration in the MPK program | Not indicated | Full year | Graduate (professional) |
| University of Windsor  Department of Kinesiology | Chronic Disease and Exercise Rehabilitation  95-461 | This course is designed to provide a broad understanding of: 1) the physiological processes involved in the development of selected chronic diseases (e.g., cardiovascular, respiratory, cancer, autoimmune) and disorders (e.g., Huntington’s disease), 2) the risk factors associated with their development and progression, where applicable, and 3) how exercise rehabilitation can be used as a tool for intervention, including past, current and emerging exercise recommendations. | Not specified | McGowan | Semester | Undergraduate |
| University of Western  School of Kinesiology | Exercise for Specific Populations: Chronic Disease  KIN 3412B | Students will learn the role of exercise in the management of chronic disease. Chronic diseases include metabolic, cardiovascular and pulmonary diseases, cancer. The implications of performing safe/effective exercise prescription and evaluation will be considered. Exercise guidelines will be examined within the context of Professional Kinesiology and clinical practice. | Registration in the Bachelor of Arts Honors Specialization in Professional Kinesiology | Mitchell | Semester | Undergraduate |
| York University  School of Kinesiology and Health Science | Exercise Therapy for Chronic Diseases  HH/KINE 4900 | An overview of the use of exercise and physical activity in the evaluation and treatment of a variety of chronic diseases and disabilities. | HH/KINE 4010 – Exercise Physiology | Not specified | Semester | Undergraduate |
| Lakehead University  School of Kinesiology | Advanced Exercise Prescription Kinesiology 5350 | Provides the theoretical foundation of advanced exercise prescription for apparently healthy individuals and for populations with chronic disease and/or related disorders, including cardiovascular diseases, obesity, cancer, diabetes, and musculoskeletal disorders. Advanced training concepts to improve health, fitness, and performance are discussed in relation to client needs. | May be taken only by students in the Graduate Diploma in Professional Kinesiology program. | Not specified | Not specified | Graduate |
| College courses (*n* = 3) | | | | | | |
| Niagara College  Exercise Science for Health and Performance Program | Exercise Prescription for Special Populations PHLT9211P | In this course the student will analyze a variety of specific conditions afflicting differing populations. This course will enable students to understand the precautions and contraindications to exercise for clients with a specific issue. In addition, the student will learn how to design and progress an appropriate program for a client who has one of the following conditions: Cardiovascular, Pulmonary, Metabolic, Musculoskeletal, Neurological, Cancer, Vision, and Hearing Impairment. | Enrollment in program | Not specified | Semester | College |
| Humber College  Exercise and Lifestyle Management Program | Therapeutic Exercise: Special Populations  ESCI 5552 | In this course, students deconstruct the American College of Sports Medicine guidelines for working with clients with special needs and/or health concerns. Students identify health conditions that influence exercise program development and analyze and modify fitness assessment and exercise program variables in order to meet the needs of these specific patient populations. | Enrollment in program | Not specified | Semester | College |
| Fanshawe College  Fitness and Health Promotion Program | Exercise for Special Populations  PHRE-3044 | By using both lecture, small group discussion and laboratory experience this course prepares students to prescribe safe and effective exercise programs for a number of special populations including individuals with a disability, children and youth, pre- and post-natal, older adults and chronic disease such as heart disease, diabetes and cancer. | Enrollment in program | Not specified | Not specified | College |

*Note.* Findings relevant as of June 2019 for the 2019 to 2020 academic year.
